# How Can We Draw the Line between Clinical Care and Medical Research

**DOI:** 10.1371/journal.pmed.0040340

**Published:** 2007-11-27

**Authors:** The PLoS Medicine Editors

## Abstract

What can editors do to help resolve misinterpretation of the boundary between clinical care and medical research?

A Policy Forum published in this month's *PLoS Medicine* highlights an issue that the *PLoS Medicine* editors have often debated: when research takes place within the context of clinical care, how can we distinguish which activities constitute care, and which research? The World Medical Association Code of Medical Ethics declares that a physician must always “act in the patient's best interest when providing medical care” [1]. Yet increasingly physicians also undertake research, which involves possibly unknown risks and benefits. These risks, and the uncertainty of benefit, might therefore conflict with a patient's best interests.

In their article, Henderson and colleagues [2] identify five key aspects of medical research that should be understood by the participants who enroll as volunteers. They specifically highlight one particular aspect, “therapeutic misconception,” which exists when “individuals do not understand that the defining purpose of clinical research is to produce generalizable knowledge.”

Misinterpretation of the boundary between research and care may have serious consequences. On the part of research participants, such misunderstanding might result in individuals enrolling in research studies believing that their physician will offer therapy that is, in the doctor's clinical judgment, the best approach for the patient personally, rather than a therapy governed by the research protocol. In another scenario, researchers who offer a treatment that is in some way different from standard or accepted practice might consider that treatment to be “off-label” prescribing, or a refinement of a surgical procedure, rather than a formal clinical trial, and fail to adequately protect the patient through ethical review and informed consent processes [3].

Given the potential for misinterpretation of the primary purpose of research, which is to collect data for the purpose of contributing to scientific knowledge, what are the requirements that must be fulfilled for involvement in a trial to be considered ethical? Many ethicists consider that, in addition to the crucial safeguards outlined in the Declaration of Helsinki [4], two additional criteria must be fulfilled (debated in [5]). Firstly, the clinical community as a whole should be uncertain as regards which of the treatments being compared is better (a scenario termed “clinical equipoise”); and secondly, completion of the trial should be likely to alter the balance of that equipoise. Importantly, Henderson et al. highlight the researchers' uncertainty with respect to risks and benefits as a key dimension of research that trial participants must understand in order to provide valid consent.

Typically, editors become involved only during the final stages of a research study, namely its preparation for dissemination. Without knowledge of specific prior circumstances, it may not be possible for an editor to see from the manuscript alone where the line should be drawn between research and care. However, editors do play a crucial role in deciding what information needs to be included in a research report, so that readers can assess the validity of the results and be assured that the procedures were ethical. But these dilemmas can be complex.

For example, a patient has been treated with a nonstandard course of therapy; the results are surprising, and the physician wishes to publish them. Was this treatment a trial (i.e., research), and should ethics committee approval and informed consent have been sought? Or is this treatment an example of “off-label” prescribing (i.e., medical care, not research)? One possible solution is to specify that when a paper is written up and submitted to a scientific journal, by definition, research has been done. Alternatively, the editor can try to gain some understanding of the clinician's original intent in treating their patient—was the treatment thought to be the best possible option for that patient at the time, or was it given specifically to produce new scientific knowledge?

Another example of the unclear boundary between research and treatment is when researchers submit a report describing the impact of a health care delivery program (for example, a report of delivering antiretroviral treatment in prisons [6]). Are such program descriptions research, requiring prior approval from a research ethics committee?

In order to resolve such cases, we often seek the advice of the *PLoS Medicine* Advisory Group on Publication Ethics [7] and the Committee on Publication Ethics (http://www.publicationethics.org.uk/); the latter body has an important role in providing a forum for journal editors from around the world to discuss such ethical dilemmas.

Since the primary purpose of health research is to produce knowledge that will ultimately improve human health, and since participants who enroll in studies expect that they will contribute to that knowledge, investigators must commit to placing their findings in the public domain [4,8]. For clinical trials, this commitment now means investigators must register basic details about every planned trial, as required by the International Committee of Medical Journal Editors [9] and many other journals, including the PLoS journals. Acceptable publicly accessible registries include ClinicalTrials.gov (http://www.clinicaltrials.gov/) and the ISRCTN (International Standard Randomized Controlled Trial Number) Web site (http://www.controlled-trials.com/isrctn/); these and other public registries are now searchable through a common portal located on the World Health Organization Web site (http://www.who.int/trialsearch/).

The editors of *PLoS Medicine* believe that open access to research findings is the most direct way of fulfilling the obligation towards participants to produce more widely available knowledge. In addition to the reporting of clinical trials, however, there is a great diversity of other types of clinical research that must be reported, and at PLoS we are committed to making these studies available in our journals. At *PLoS ONE,* the peer-review process focuses on the technical quality of the work done; *PLoS ONE* is committed to providing a venue for publication of the results of all correctly conducted and reported trials, irrespective of outcome.

In addition, PLoS has just launched the PLoS Hub for Clinical Trials (http://clinicaltrials.ploshubs.org/); this site provides a single point of access to the results of trials, and other types of research that are relevant to trials, from multiple PLoS journals, including *PLoS ONE.* This site, and future PLoS Hubs, aims to go beyond the traditional conception of a journal Web site. Tools are available for users to rate, annotate, and discuss published articles. Open access ensures that research findings are available to patients and the public to read and reuse freely. We hope that the availability of the findings of clinical studies will help to increase public understanding of research, and to provide the public with ways in which they can comment directly on the work that has been done.
